# Does Regular Dancing Improve Static Balance?

**DOI:** 10.3390/ijerph18105056

**Published:** 2021-05-11

**Authors:** Przemysław Stawicki, Agnieszka Wareńczak, Przemysław Lisiński

**Affiliations:** Department of Rehabilitation and Physiotherapy, University of Medical Sciences, 28 Czerwca 1956 Str., No 135/147, 60-545 Poznań, Poland; pabstawicki@gmail.com (P.S.); plisinski@vp.pl (P.L.)

**Keywords:** balance, dance, biomechanics, woman’s health, posturography

## Abstract

The aim of the study was to compare the static balance of dancers and non-dancers in a bipedal and unipedal stance. Twenty-three female dancers (mean age: 21.3 ± 1.7) and 24 age and sex-matched subjects (mean age 22.3 ± 1.0) participated in this study. A force platform was used to assess balance. The tests on the balance platform were performed in several positions with different foot placement, such as normal standing (NS) eyes open and eyes closed positions, semi-tandem position (ST), tandem position (TP), and one-leg standing (1L) eyes open and eyes closed position. Significant differences in balance between the dancers and the control group, especially in the tandem position and one-leg standing position with eyes closed were found. We observed higher results for the velocity of the COP in the frontal plane in the TP with a dominant limb in front (*p* = 0.04) and higher results for the velocity of the COP in the frontal plane (*p* = 0.01) and in the sagittal plane (*p* < 0.01) in the TP with a dominant limb in front in the control group. We also observed significant differences between groups in the mean velocity of COP sway in the sagittal plane in the 1 L position with eyes closed (*p* = 0.04). We concluded that dancing regularly for several years improves static balance.

## 1. Introduction

Postural stability (balance) is defined as the ability to maintain the body’s center of mass (COM) within its base of support (BOS) [1]. The inputs from the visual, somatosensory, and vestibular systems must be integrated and then processed within the central nervous system into efferent (motor) commands to activate the appropriate motor responses necessary for maintaining balance [2,3]. As suggested by Montemurro et al., cortical frontal eye field also helps in maintaining posture [4].

Of the three sensory systems governing postural control, the proprioceptive inputs are thought to have the most significant influence in detecting body sway [5]. The data show that proprioception can be evaluated by various methods, including the joint position sense (JPS) test [6]. However, the assessment of balance is usually based on an analysis of body sway. Paillard and Noé [7] indicated that postural control could also be considered by measuring the movement of the body segments, measuring electromyographic activities, and evaluating the contribution of various sensory information. Monteiro-Junior [8] reports that a force plate is considered a gold standard tool for assessing body balance. Quantitative posturography includes a set of standing balance test protocols. It provides an objective measurement of postural sway and shows changes in the center of foot pressure (COP) [9]. As suggested by Mancini and Horak [10], comprehensive clinical assessment of balance is important for diagnostic and therapeutic reasons in clinical practice.

Body balance also depends on several external and internal factors. An individual’s ability to balance depends on genetics, age, center of mass positioning, the area of support, emotional state, strength, coordination, flexibility, frequency of participation in motor activities, and training status and visual control [11]. Among the many activities, dance is mentioned as the one that improves balance. Some researchers indicated the effects of balance training on postural control and mobility in older adults [12,13]. Older people can significantly improve their aerobic capacity, lower body muscle endurance, strength and flexibility, physical reaction time, agility, and gait through dancing [13]. Dancing is also a type of physical activity that reduces the prevalence of falls and cardiovascular health risks [14].

As we know, postural balance (PB) is an important component skill for professional dancers [15]. Janura et al. [16] suggested that dance performance is a complex act, with many elements, including strength, balance, flexibility, and endurance. The data show that the dancers’ skill depends largely on effective technical training, with elements of good posture and balance, and requires the codification of sensory input to build mental representations of the action to be performed [17]. The question is whether closing the eyes causes the same deterioration of balance in dancers and a control group. Bruyneel et al. [18] indicated the importance of vision for maintaining balance in ballet dancers. Hutt and Redding [19] suggested that dancers may use visual input as the dominant mechanism to maintain balance when their eyes are open, yet acutely shift to more proprioceptive strategies when their eyes are closed.

According to the other authors, balance assessment can be performed in static and dynamic conditions [20]. Many studies have focused on assessing ballet dancers [16,17,21] and assessing static and postural stability in dancers and non-dancers in neutral foot positions or on one leg position [16,22]. In our study, we wanted to check if dancing three times a week for a minimum of seven years affects the maintenance of static balance in various bases of support (in various feet positions).

It is still unclear whether postural control in dancers is different than in untrained subjects in static positions. Therefore, the aim of this study was to assess whether there are differences in postural control in static conditions between dancers who were training for a minimum of 7 years and non-dancers, and if these differences are associated with different bases of support and vision. The hypothesis of the study was that females who regularly dance modern and classical dance demonstrate better static balance than participants of similar age and overall health.

## 2. Materials and Methods

### 2.1. Participants

A study included 47 female students of Poznan University of Medical Sciences, 23 dancers, who composed a study group, and 24 students, constituting a control group. The mean age in the dancer group was 21.3 years (SD = 1.7) and 22.3 (SD = 1.0) in the control group. No significant differences in age were found between the control and study groups. Participants from the study group had been dancing for a minimum of 7 years. The average dancing time during the week was 6 to 7 h. No significant differences in height (*p* = 0.19), weight (*p* = 0.47), or BMI (*p* = 0.40) were found between the study and control groups (Table 1). Participants were evaluated only once and were informed about the aims and methodology. They also provided written consent to participate in the project. The characteristics of the study population are shown in Table 1.

The following inclusion criteria for both groups were used: age from 20 to 24 years and the participants’ written consent. People recruited to the study group had performed training (modern and/or classical dance) a minimum of three times a week for a minimum of seven years. The participants recruited to the control group did not dance. They participated only in physical education classes organized by the universities, 1.5 h per week. The exclusion criteria for both groups were: injuries and surgical treatments of the lower limbs, severe systemic diseases and severe neurological disorders, the presence of pain anywhere in the body, or feet deformities. The information necessary to qualify participants for the study was obtained from an interview before the participant’s testing.

The study was conducted in compliance with the Declaration of Helsinki and with the approval of the Bioethics Committee of Poznan University of Medical Sciences (reference number 1053/19). Data were anonymized before analysis.

### 2.2. Experimental Procedures and Instruments

The static balance tests, including body sway measurements, were performed on a Metitur Good Balance platform. The Good Balance system^®^ consists of an equilateral triangular force platform connected to a computer [23]. Force platform balance tests provide valid information on postural control. The literature indicates the reliability of the Metitur force platform in the assessment of balance [23,24]. This force platform with integrated digital electronics and wireless data transmission allows the analysis of many parameters characterizing balance. The system produces a dimensional curve based on the force signals showing the amount and characteristics of postural sway throughout the registration period. The results are then calculated as absolute units or in units adjusted for body height (our analysis was based on units adjusted for body height). The software also calculates several variables describing the quantitative aspects of body sway. In our study we analyzed the following parameters:

- MV Y (mm/s): the mean velocity of the sway of the center of feet pressure (COP) in the sagittal plane,
- MV X (mm/s): the mean velocity of the sway of the COP in the frontal plane,
- Spectrum Y (Hz, mm): the value of the middle of the spectrum in the saggital plane, the spectrum was characterized by two codependent variables, such as frequency (Hz) and excursion (mm),
- Spectrum X (Hz, mm): the value of the middle of the spectrum in the frontal plane (frequency and excursion).

Spectral analyses of COP sway are usually performed with algorithms based on Fourier transforms [7]. The centre of the spectrum is based on the frequency/amplitude analysis included in the Good Balance software. The movement of the center of pressure is first split into the anterioposterior (Y) and medial/lateral (X) directions. Next, fast Fourier transformation (FFT) analysis is used to calculate the frequency/amplitude spectrum of the signal separately in these two directions. One of the outcomes of the FFT analyses is the centre of the spectrum, this means, in practice, the frequency and corresponding amplitude that splits the spectrum into two parts, each including the same amount of energy. So it can be seen as the “most typical frequency and corresponding amplitude in the spectrum”.

**Figure 1:**
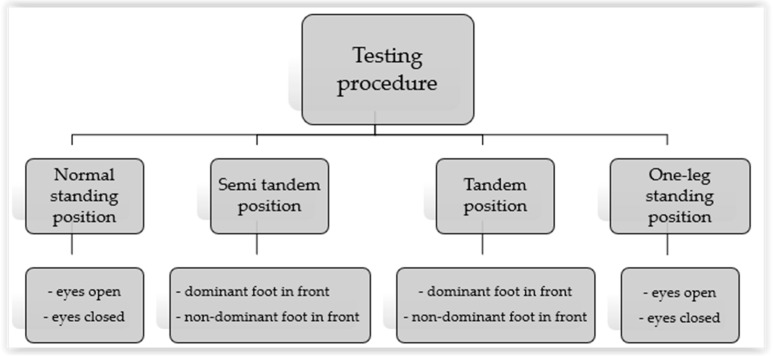
Testing procedures.

The tests performed on the force platform were conducted in several positions with different foot placements (Figure 1):

- Normal standing with eyes open (NS EO) and eyes closed (NS EC) positions
- Semi-tandem position (ST)
- Tandem position (TP)
- One-leg standing position (1L)

The participants performed all measurements barefoot. They were asked to maintain a fairly upright position. During the test with opened eyes, participants were asked to look straight ahead at a visual reference point.

#### 2.2.1. Normal Standing Position (NS)

**Figure 2:**
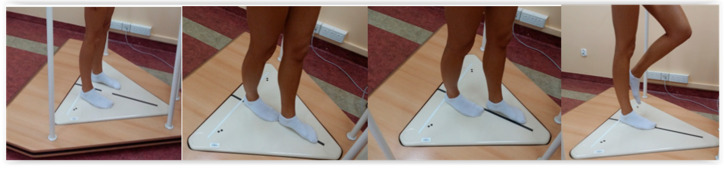
Testing procedure (from the left side): normal standing position, tandem position, semi-tandem position, one-leg standing position.

In the normal standing position with eyes open and eyes closed, the feet were placed 20 cm apart (distance was measured from the center of each heel) and parallel to each other (Figure 2). The duration of the tests was 30 s each [25].

#### 2.2.2. Semi-Tandem Position (ST)

In the STP, the left or right foot was placed in front of the other, but the feet were placed on both sides of a line that divided the platform into two parts. The tests were performed with the dominant foot in front (SDF) and with the non-dominant foot in front (SNF), separately by 20 s (Figure 2).

#### 2.2.3. Tandem Position (TP)

During the TP test, one foot was placed directly ahead of the other (Figure 2). The length of the exercise was 20 s. Similarly to the semi-tandem test, the patients were examined with the dominant (TDF) and non-dominant (TNF) foot in front.

#### 2.2.4. One-Leg Standing Position (1L)

When testing the leg standing position on the balance platform, the patients were asked to raise a non-dominant foot to the supporting leg’s mid-calf level but not touch the loaded limb (Figure 2). To obtain an objective result, the participant must independently maintain the position. We stopped the test, and the trial was incomplete when the participant had to use the arms (touched the handrail) or used the raised foot (touched the floor). These results were not further analyzed. Participants were examined when standing on a dominant limb with eyes open (1EO) and eyes closed (1EC). Time of test was 20 s.

### 2.3. Statistical Analysis

Data were analyzed with Statistica software version 13.1. Demographic data and clinical characteristics are presented as means, standard deviations (SD), median, minimum (min), and maximum (max). The Shapiro–Wilk test was used to assess the normality of the distributions in the test score. To compare the differences between the THR group and the control group, we applied the independent t-test, the Welch test, or the non-parametric U Mann–Whitney test, depending on which test assumptions were met. *p*-values less than 0.05 were considered statistically significant.

## 3. Results

### 3.1. Normal Standing Position (NS)

During the static test in the NS eyes open and NS eyes closed position, the results obtained in the sagittal plane, such as the middle of the spectrum, differed significantly between the study and control groups (Table 2). The frequency of the middle of the spectrum was higher in the study group. No significant differences between the mean velocity of COP displacement in both planes were found in either group.

### 3.2. Semi-Tandem Position (ST)

In the semi-tandem positions (the dominant foot in front and the non-dominant foot in front) only the results of the middle of the spectrum in the frontal plane were statistically significant (Table 3).

### 3.3. Tandem Position (TP)

Higher mean and median results for the velocity of the COP in the frontal plane in the tandem position with the dominant limb in front were observed in the control group. The median of the mean velocity of COP sway in the dancer group was 1.05 mm/slower than that in the control group. Furthermore, the results of the middle of the spectrum (frequency and excursion) in a frontal plane differed significantly between the study and control groups. In the tandem position with a non-dominant leg in front, additionally, we observed higher mean and median results for the velocity of the COP in the sagittal plane (*p* < 0.01). All results are presented in Table 4.

### 3.4. One-Leg Standing Position (1L)

No significant differences in the values of the measured parameters were found during a one-leg standing test with eyes open in both groups (Table 5). Two subjects from the control group did not complete this trial. We observed significant differences in one leg standing position with eyes closed. The median of the mean velocity of COP sway in the sagittal plane in the control group was 4.7 mm/s higher than that in the study group. Six subjects from the control group and two dancers did not complete this trial.

## 4. Discussion

Due to its specificity, dance is a unique form of training that contains balance training, endurance training, strength training, and flexibility training in varying amounts. Numerous studies have demonstrated that dancing helps develop coordination skills that affect improved balance [19,26,27]. In our research, we assessed whether practicing dance a minimum of three times a week for several years improves static balance compared to people with low physical activity. Many investigations in the literature describe the impact of ballet on balance [17,19,28]. Contrary to the above statement, dancers qualified for our research had modern and/or classical dance experience. We observed many differences between our study group and the control group, indicating the better control of static balance with eyes open and closed of the dancers. The other studies also demonstrate the impact that dance activity and associated movements have on enhancing static balance [11].

Some results indicate that dancers’ postural control is strongly dependent on the availability of visual information [29,30]. Closing the eyes will always deteriorate the balance, even in healthy and trained people [31]. The question is whether closing the eyes causes the same deterioration of balance in the dancers and the control group. In our research there were differences in maintaining posture both in the NS eyes open and closed position between dancers and non-dancers. The frequency of the middle of the spectrum in the sagittal plane differed significantly between groups. The frequency of COP displacement was higher in the dancer group, indicating a more effective postural response to body sway in dancers than non-dancers, independently of the availability of visual information. Pérez et al. achieved different results. The authors observed a significantly greater balance ability in the dancer group, compared to non-dancers, only in tasks with eyes open. The authors supposed that these results may be due to dancers’ increased specialization in tasks where postural control is regulated by visual feedback [32]. Kuczyński et al. [33] suggested that the postural control of dancers and non-dancers appears to be similar, although dancing seems to facilitate an increased level of automatic control in the antero-posterior plane [33]. Michalska et al. [29] showed that professional ballet dancers demonstrated higher values of postural sway characteristics in comparison to non-trainees while performing simple motor tasks. Authors suggested that his might be a sign of a higher capacity of the postural system to deal with postural instability in dancers [29]. In general, the authors agreed on the better balance of dancers, but most studies indicate the influence of eyesight on maintaining balance in this group.

We found significant differences in measured parameters during non-symmetric leg loading tests (semi-tandem and tandem position). In semi-tandem positions (the dominant foot in front and the non-dominant foot in front), the range of the middle of the spectrum in the frontal plane was higher in the non-dancer group. This means that they displayed a greater range of body sway related to poorer balance. Furthermore, in the tandem position (TP), the dance group held a more stable position than the control group. In the tandem position with the non-dominant leg in front, we observed a higher mean velocity of COP displacement in the sagittal plane in the non-dancer group. In both tandem positions, we observed a significantly higher mean velocity of COP sway in the frontal plane in the control group than the dancer group. Additionally, in the dancer group during the tandem with dominant foot in front test, we observed better results characterizing the spectrum. They achieved a higher frequency of COP sway with a smaller range of body sway in the frontal plane. These two parameters indicate better postural control reactions in the dancer group compared to the non-dancer group. The literature review did not find many studies that applied the semi-tandem and tandem positions for assessing balance in dancers using a study method analogous to ours [34]. Many researchers assessed the balance in a neutral foot position or the one leg standing position. Harmon et al. [22] evaluated balance in the ballet position (first ballet position in which heels were touching and feet were externally rotated to 140°, and sixth ballet position in which heels were spaced 10 cm apart and forward parallel) of the feet using a Y-balance test and motor control test and suggested that superior balance and motor control in dancers may be limited to less innate dance-specific foot positions. Casabona et al. [34] assessed professional ballet dancers and non-dancers in different foot positions, such as common stances (with varying heel spaces), duck (feet in extra rotation, heels together, and an opening angle of 140°), and tandem position. Contrary to our observations, the authors did not find any significant differences in the tandem position. The authors observed significant differences only for the stances with a foot position familiar to the dancers. They concluded that the benefit from classical ballet is limited to a specific foot configuration, regardless of the level of stance difficulty or the component of postural control [34].

We did not observe significant differences between the groups in the one-leg standing position with eyes open (1L). Despite the lack of statistically significant differences, we observed increased postural sway in both planes in the control group compared to the dancer group. Additionally, we found a significantly greater mean velocity of COP sways in the sagittal plane in the control group in the one-leg standing position with eyes closed. It is worth mentioning that two participants from the study group did not complete the one leg standing test with eyes open, and six participants from this group did not complete these tests with eyes closed, which indicates the better balance of the dancers. The results obtained by the other authors in this position were different. Bharnuke et al. [35], who was assessing Indian classical dancers, showed that the dancers demonstrated the better balance in a variety of tasks, including tasks performed on a wide base of support with eyes open and with eyes closed and during single limb stance with eyes open and with eyes closed [35]. Crotts et al. [36] noticed that dancers could maintain a stable position for longer during a single-leg stance with eyes closed than non-dancers. This may indicate a greater efficiency of the proprioceptive system in dancers. During the same trial with eyes open, dancers had similar results as a control group, so the authors suggested that dancers’ postural stability is less dependent on visual information [36]. Kilroy et al. [11] concluded that seven or more years of dance experience increased balance ability when performing a single-leg stance and showed that dancers consistently demonstrated less deviation in the AP (anterior–posterior) and ML (medial–lateral) ground reaction forces during barefoot and shod conditions compared to non-dancers. Costa de Mello and colleagues [28] suggested that professional ballet dancers show greater visual dependency for balance adjustment with reduced influence of the supporting base on postural sway. Janura et al. [16], who compare ballet dancers and non-dancers, presented different outcomes. The authors found that, for the unipedal stance with eyes open and eyes closed, the sway and the velocity characterizing the COP movement in the dancers and the control group were generally comparable.

Our research had several limitations. We did not perform a sample size calculation before starting the test, so it can be considered a pilot study. Another research limitation is that the semi-tandem and tandem tests were performed with eyes open only. Only women participated in the study, which may also be a limitation of our research.

## 5. Conclusions

The present study results support the hypothesis that young females who dance regularly (an average three times a week, 6–7 h for a week) for a minimum of 7 years demonstrate better static balance than females, who are age and overall health matched, who do not regularly participate in sports or dance activities. The dancers achieved significantly better results characterizing COP displacement during NS position with eyes open and closed, and the ST, TP, and 1L position with eyes closed. We concluded that dancing regularly for several years (although it is a dynamic activity) improves static balance. As dance improves balance in young people, it is worth using this form of training to prevent poor balance in different age groups.

## Figures and Tables

**Table 1:** Demographic data of participants.

	Dancers			Control Group			
Variable	Mean ± SD	Median	Min-Max	Mean ± SD	Median	Min-Max	*p*
weight (kg)	56.52 ± 7.59	54.00	45.00–80.00	58.48 ± 6.80	57.00	47.00–73.00	0.19 *
height (cm)	165.70 ± 5.86	167.00	151.00–176.00	167.00 ± 6.33	167.50	155.00–180.00	0.47
BMI	20.59 ± 2.52	20.06	15.39–28.69	20.94 ± 1.93	20.89	17.47–25.39	0.40 *

*t*-student test, * Mann–Whitney test.

**Table 2:** Comparison of results obtained with normal standing, eyes open (NS EO) and eyes closed (NS EC) positions in the dancers and control groups.

		Dancers Group			Control Group			
	Variable	Mean ± SD	Median	Min-Max	Mean ± SD	Median	Min-Max	*p*-Value
NS EO	MV Y (mm/s)	4.15 ± 1.12	3.80	2.70–6.50	4.54 ± 0.75	4.70	3.20–5.8	0.07 *
	MV X (mm/s)	2.43 ± 0.96	2.20	1.20–4.60	2.86 ± 0.90	3.05	1.10–4.50	0.08 *
	Spectrum Y (Hz)	0.436 ± 0.173	0.398	0.142–0.805	0.347 ± 0.121	0.352	0.120–0.540	0.04
	Spectrum Y (mm)	0.322 ± 0.030	0.321	0.247–0.382	0.327 ± 0.033	0.322	0.282–0.402	0.61
	Spectrum X (Hz)	0.282 ± 0.156	0.156	0.124–0.758	0.299 ± 0.146	0.244	0.135–0.691	0.54 *
	Spectrum X (mm)	0.080 ± 0.054	0.069	0.041–0.307	0.078 ± 0.024	0.080	0.038–0.127	0.35 *
NS EC	MV Y (mm/s)	6.69 ± 2.09	6.30	4.10–12.30	6.80 ± 1.91	6.40	4.20–11.00	0.77 *
	MV X (mm/s)	3.02 ± 0.90	3.00	1.80–6.00	3.30 ± 1.08	3.20	1.70–5.90	0.31 *
	Spectrum Y (Hz)	0.460 ± 0.127	0.440	0.263–0.712	0.375 ± 0.123	0.372	0.201–0.672	0.02
	Spectrum Y (mm)	0.354 ± 0.041	0.346	0.298–0.447	0.350 ± 0.070	0.349	0.108–0.473	0.90 *
	Spectrum X (Hz)	0.386 ± 0.176	0.365	0.183–0.867	0.335 ± 0.147	0.292	0.183–0.636	0.23 *
	Spectrum X (mm)	0.078 ± 0.020	0.079	0.039–0.115	0.091 ± 0.036	0.083	0.040–0.177	0.11

MV, the mean velocity of the sway; *t*-student test, * Mann–Whitney test.

**Table 3:** Comparison of results obtained in the semi-tandem position with the dominant foot in front (SDF) and with the non-dominant foot in front (SNF) in the dancer and control groups.

		Dancers Group			Control Group			
	Variable	Mean ± SD	Median	Min-Max	Mean ± SD	Median	Min-Max	*p*-Value
SDF	MV Y (mm/s)	6.87 ± 1.37	6.80	3.50–9.20	7.26 ± 1.64	6.65	4.70–11.10	0.38
	MV X (mm/s)	6.62 ± 1.32	6.50	4.40–9.30	7.47 ± 1.70	7.20	4.20–11.90	0.06
	Spectrum Y (Hz)	0.536 ± 0.199	0.527	0.242–0.949	0.440 ± 0.181	0.422	0.209–1.066	0.07 *
	Spectrum Y (mm)	0.403 ± 0.044	0.398	0.335–0.502	0.409 ± 0.053	0.403	0.308–0.544	0.70
	Spectrum X (Hz)	0.312 ± 0.117	0.305	0.133–0.554	0.292 ± 0.096	0.298	0.135–0.533	0.51
	Spectrum X (mm)	0.219 ± 0.044	0.214	0.145–0.347	0.261 ± 0.060	0.261	0.142–0.403	0.01
SNF	MV Y (mm/s)	6.51 ± 1.52	6.20	3.20–9.50	7.19 ± 2.08	6.50	4.90–13.50	0.32 *
	MV X (mm/s)	6.67 ± 1.09	6.50	4.70–8.60	7.49 ± 1.66	7.20	4.90–10.70	0.05
	Spectrum Y (Hz)	0.454 ± 0.222	0.379	0.200–1.120	0.459 ± 0.202	0.410	0.192–1.044	0.66 *
	Spectrum Y (mm)	0.407 ± 0.055	0.409	0.307–0.515	0.415 ± 0.081	0.394	0.288–0.632	0.76 *
	Spectrum X (Hz)	0.299 ± 0.100	0.283	0.184–0.471	0.323 ± 0.115	0.294	0.181–0.593	0.63 *
	Spectrum X (mm)	0.224 ± 0.042	0.218	0.158–0.321	0.259 ± 0.065	0.256	0.152–0.374	0.03

*t*-student test, * Mann–Whitney test.

**Table 4:** Comparison of results obtained in the tandem position with the dominant foot in front (TDF) and with the non-dominant foot in front (TNF) in the dancer and control groups.

		Dancers Group			Control Group			
	Variable	Mean ± SD	Median	Min-Max	Mean ± SD	Median	Min-Max	*p*-Value
TDF	MV Y (mm/s)	8.97 ± 1.82	8.90	6.10–12.90	10.34 ± 2.41	9.70	7.70–16.80	0.07 *
	MV X (mm/s)	8.76 ± 1.60	8.40	6.10–13.40	10.00 ± 2.46	9.45	7.50–18.90	0.04 *
	Spectrum Y (Hz)	0.475 ± 0.173	0.474	0.216–0.972	0.503 ± 0.239	0.474	0.177–1.150	0.77 *
	Spectrum Y (mm)	0.329 ± 0.076	0.327	0.232–0.498	0.332 ± 0.061	0.321	0.248–0.482	0.86
	Spectrum X (Hz)	0.366 ± 0.124	0.347	0.180–0.655	0.276 ± 0.088	0.266	0.150–0.450	0.01
	Spectrum X (mm)	0.269 ± 0.037	0.271	0.206–0.350	0.329 ± 0.060	0.322	0.227–0.540	<0.01 *
TNF	MV Y (mm/s)	8.57 ± 2.00	7.80	6.20–13.40	10.35 ± 2.31	10.25	7.20–17.10	<0.01 *
	MV X (mm/s)	8.44 ± 2.21	8.10	4.60–15.00	10.27 ± 3.32	9.60	6.80–23.80	0.01 *
	Spectrum Y (Hz)	0.417 ± 0.188	0.343	0.166–0.837	0.471 ± 0.156	0.459	0.267–0.837	0.28
	Spectrum Y (mm)	0.300 ± 0.093	0.290	0.025–0.489	0.330 ± 0.057	0.330	0.254–0.478	0.18
	Spectrum X (Hz)	0.340 ± 0.113	0.326	0.161–0.561	0.362 ± 0.134	0.352	0.161–0.599	0.55
	Spectrum X (mm)	0.270 ± 0.051	0.265	0.177–0.373	0.323 ± 0.086	0.312	0.191–0.623	0.02 *

*t*-student test, * Mann–Whitney test.

**Table 5:** Comparison of results obtained in the one-leg standing position with eyes open (1L EO) and eyes closed (1L EC) in the dancer and control groups.

		Dancers Group			Control Group			
	Variable	Mean ± SD	Median	Min-Max	Mean ± SD	Median	Min-Max	*p*
1L EO	MV Y mm/s	14.50 ± 4.11	13.60	8.90–24.30	16.03 ± 3.26	15.40	11.60–24.30	0.18
	MV X mm/s	16.00 ± 3.10	15.70	10.70–25.20	17.27 ± 3.64	17.05	10.80–24.70	0.22
	Spectrum Y (Hz)	0.400 ± 0.159	0.401	0.217–0.756	0.470 ± 0.164	0.443	0.285–0.898	0.41 *
	Spectrum Y (mm)	0.597 ± 0.108	0.595	0.445–0.815	0.608 ± 0.057	0.608	0.475–0.720	0.67
	Spectrum X (Hz)	0.693 ± 0.216	0.667	0.388–1.117	0.638 ± 0.195	0.610	0.328–1.058	0.37
	Spectrum X (mm)	0.424 ± 0.054	0.421	0.322–0.540	0.465 ± 0.071	0.442	0.366–0.616	0.08 *
1L EC	MV Y mm/s	33.60 ± 11.43	32.00	14.10–70.80	37.67 ± 7.39	36.70	23.90–53.80	0.04 *
	MV X mm/s	36.88 ± 10.15	37.60	19.20–55.30	38.47 ± 7.90	38.65	25.30–60.10	0.59
	Spectrum Y (Hz)	0.435 ± 0.087	0.424	0.272–0.575	0.455 ± 0.150	0.447	0.250–0.723	0.62
	Spectrum Y (mm)	1.060 ± 0.340	1.019	0.612–2.082	1.180 ± 0.340	1.190	0.712–2.276	0.15 *
	Spectrum X (Hz)	0.564 ± 0.083	0.556	0.407–0.746	0.564 ± 0.082	0.554	0.436–0.750	0.99
	Spectrum X (mm)	0.962 ± 0.277	0.895	0.479–1.565	0.943 ± 0.148	0.952	0.664–1.238	0.78

*t*-student test, * Mann–Whitney test.

## Data Availability

The data presented in this study are available on request from the first author. The data are not publicly available due to ethical restrictions.
